# Weed abundance is positively correlated with native plant diversity in grasslands of southern Australia

**DOI:** 10.1371/journal.pone.0178681

**Published:** 2017-06-01

**Authors:** Irene Martín-Forés, Greg R. Guerin, Andrew J. Lowe

**Affiliations:** 1 Department of Ecology, Complutense University of Madrid, Madrid, Spain; 2 Terrestrial Ecosystem Research Network, School of Biological Sciences, The University of Adelaide, Adelaide, Australia; 3 Environment Institute, School of Biological Sciences, The University of Adelaide, Adelaide, Australia; Chinese Academy of Forestry, CHINA

## Abstract

Weeds are commonly considered a threat to biodiversity, yet interactions between native and exotic species in grasslands are poorly understood and reported results vary depending on the spatial scale of study, the factors controlled for and the response variables analysed. We tested whether weed presence and abundance is related to declines in biodiversity in Australian grasslands. We employed existing field data from 241 plots along a disturbance gradient and correlated species richness, cover and Shannon diversity for natives and exotics, controlling for seasonal rainfall, climatic gradients and nutrient status. We found no negative relationships in terms of emergent diversity metrics and occupation of space, indeed, many positive relationships were revealed. When split by land-use, differences were found along the disturbance gradient. In high-moderately disturbed grasslands associated with land-uses such as cropping and modified pastures, positive associations were enhanced. Tolerance and facilitation mechanisms may be involved, such as complementary roles through different life history strategies: the exotic flora was dominated mainly by annual grasses and herbs whereas the native flora represented more diverse growth-forms with a higher proportion of perennials. The positive relationships existing between native and exotic plant species in high-moderately disturbed grasslands of South Australia are most likely due to facilitation through different strategies in occupation of space given that the effect of habitat suitability was controlled for by including environmental and disturbance factors. Consequently, although particular weeds may negatively impact biodiversity, this cannot be generalised and management focusing on general weed eradication in grasslands might be ineffectual.

## Introduction

Negative connotations are commonly invoked by the terms weed, alien and exotic when referring to biodiversity assets. Indeed, in most conservation programs, exotic plant species are regarded as injurious competitors responsible for damaging ecosystems [1,2] and threatening native biodiversity [3,4]. Negative connotations associated with exotic plant species are reflected in a publication bias in the ecological literature towards studies focusing on biological invasions by exotic species as a harmful process with negative impacts, a current focus of ecological research [5].

Undoubtedly, demonstrating the damage that exotic species can cause to ecology [6,7], economy [8,9] and society [10] is important. However, such studies tend to focus on problematic weeds with visible negative impacts on native biodiversity. Studies regarding the impacts of invasive native species [11], or those focusing on the possible benefits that weeds can provide, have received less attention [12–14].

In the last decade, the bias in the literature towards negative impacts from exotics has started to change. In terms of species richness, recent studies have illustrated that the relationship between native and exotic is not always negative (a fact known as ‘the invasion paradox’ [15]); instead, it depends on the scale of study, being negative at small spatial scales (<10m^2^) and positive at large spatial scales (>10m^2^). Similarly, some authors recently highlighted that certain invasive species can act as keystone elements enhancing the survival of local endemics and that in some cases invasive species eradication programs aiming to re-establish the original vegetation might result in population bottlenecks, local extinction [14,16] and cascading effects across trophic levels [12,17] or other ecosystem components [18]. At the ecological community level, there is no consensus on the general impact of exotic species diversity.

Grasslands are mainly semi-natural ecosystems, often with agro-silvopastoral management and/or extensive grazing by livestock. Grasslands from Mediterranean climate regions are one of the most diverse ecosystems in the world and therefore considered biodiversity hotspots and targets for conservation efforts. The frequency of weeds in Mediterranean ecosystems is considerable, although it varies among regions [19,20]. Invasion by exotic species is frequently cited as a key threat to remnant grasslands [21–23], leading to metrics associated with weed diversity being routinely employed as general condition indicators (e.g. [22]). Therefore, it is highly relevant to the assessment and management of these systems to better understand the influence of weed diversity on native diversity.

Experimental manipulation of native and exotic diversity as well as *post hoc* assessment of restoration plots in grassy ecosystems have found negative effects when exotics were present, including decreased native abundance, richness, growth and regeneration [21,23,24] and altered species composition [24]. These negative relationships were backed up by a global analysis of grassland plots, which found that exotic richness and cover negatively correlated with native richness [25].

Nevertheless, positive associations between exotics and natives have also been reported, especially in the Mediterranean Biome. Positive correlation between native and exotic richness were reported in Chilean grasslands [26] and scrub communities [27]. Similar relationships were also documented for post-fire vegetation communities in Californian chaparral [28,29] and grasslands [30]. It was also recently reported that exotic species appear to play a complementary role to native species recovery in community assembly along a secondary successional gradient in Chilean Mediterranean grasslands [31]. All the previous studies were conducted at small spatial scales (between 0.25m^2^ and 1m^2^) and only considered species richness metrics and not cover nor abundance or Shannon diversity. Unfortunately, studies in the Mediterranean Biome focused on large-scale processes have been underrepresented (but see for example [32]). Likewise, studies taking into account species abundance, species cover and Shannon diversity are uncommon, especially at larger-spatial sampling scales, even though the inclusion of these variables could detect negative relationships between native and exotic species [29].

Less clear-cut cases have also been reported. For example, weak relationships between native richness and exotic cover were found in temperate grassy woodlands when accounting for the positive, nonlinear relationship of both with rainfall, and the relationship was positive at one study site and negative at another, a result possibly relating to contrasting disturbance histories and responses [33]. Controlling for environmental conditions and land-use is of key importance when assessing relationships between natives and exotics at broad scale. In fact, when re-analysing data from large-spatial scale studies by including factors such as climate and degree of disturbance, the relationship between native and exotic species could become negative [15].

A recurring lesson across these studies is that relative abundance, usually measured as cover, is needed to fully interpret exotic—native diversity interactions [25], because at low exotic richness overall exotic cover ranges from very low to very high, depending on the dominance of individual species. Basic empirical research is still needed to build a more general picture of interactions between exotic and native richness, diversity and cover, and elucidating these relationships can inform on-ground management of threatened grassland ecosystems, for example whether reducing exotic species diversity and cover, regardless of species identity, is beneficial.

In this study, we tested whether there is a negative association between the presence of exotic species and native plant biodiversity in southern Australian grasslands associated with different land-uses, along a disturbance gradient. We hypothesised that, in general, native biodiversity would not be diminished by exotic species richness in these grasslands because the exotic species were already adapted to these anthropogenic activities in their region of origin (‘eco-evolutionary experience’ [34]). Due to this co-evolution of the exotic species with human activity and agrarian practices, their establishment in disturbed grasslands might soften the harsh conditions, favouring the development of native species. The different evolutionary contexts of both species groups suggest they employ different strategies to occupy ecological and physical space, so we expected to find positive correlations between exotic and native species cover. Furthermore, we expected that the hypothesised lack of competition between exotic and native species would be accentuated for agrarian land-uses (cropping and grazing) because these are typical land-uses in which the exotic species existed in their region of origin, mainly the Mediterranean Basin [35–37]. We examined overall effects of richness, diversity and cover in both species groups and controlled for the influence of environmental setting and climate on these parameters. Components of these systems are considered a threatened ecological community [22] and so the outcome of this study is relevant for management strategies as well as providing data towards more general questions around exotic diversity and its impacts at ecological community level.

## Materials and methods

### Dataset

We employed data originating from the Biological Survey of South Australia (BSSA) [38,39]. The BSSA consists of both a method and a series of systematic surveys conducted across the state of South Australia to provide a broad baseline inventory of the State’s flora and fauna and to document the diversity of native vegetation communities and areas of high biodiversity [40,41], while some surveys were conducted for particular purposes such as habitat mapping. We selected a set of surveys that specifically targeted grasslands or local regions where grasslands are the predominant vegetation type (Burra Hills, Lofty Block Grasslands, Pygmy Blue Tongue Project, Temperate Grasslands—WWF [42,43]).

Survey sites were typically but not exclusively located within vegetation determined *a priori* to be 'native', although much of the semi-natural grassland in the study region occurs on private land that has been grazed or modified in some way [22]. Remnant grasslands also exist on public land such as Crown Land, rail and road reserves and local government reserves. All surveys were located within the Mediterranean climate zone of the State (more arid grasslands were not included), a region that is used intensively for agriculture.

**Fig 1:**
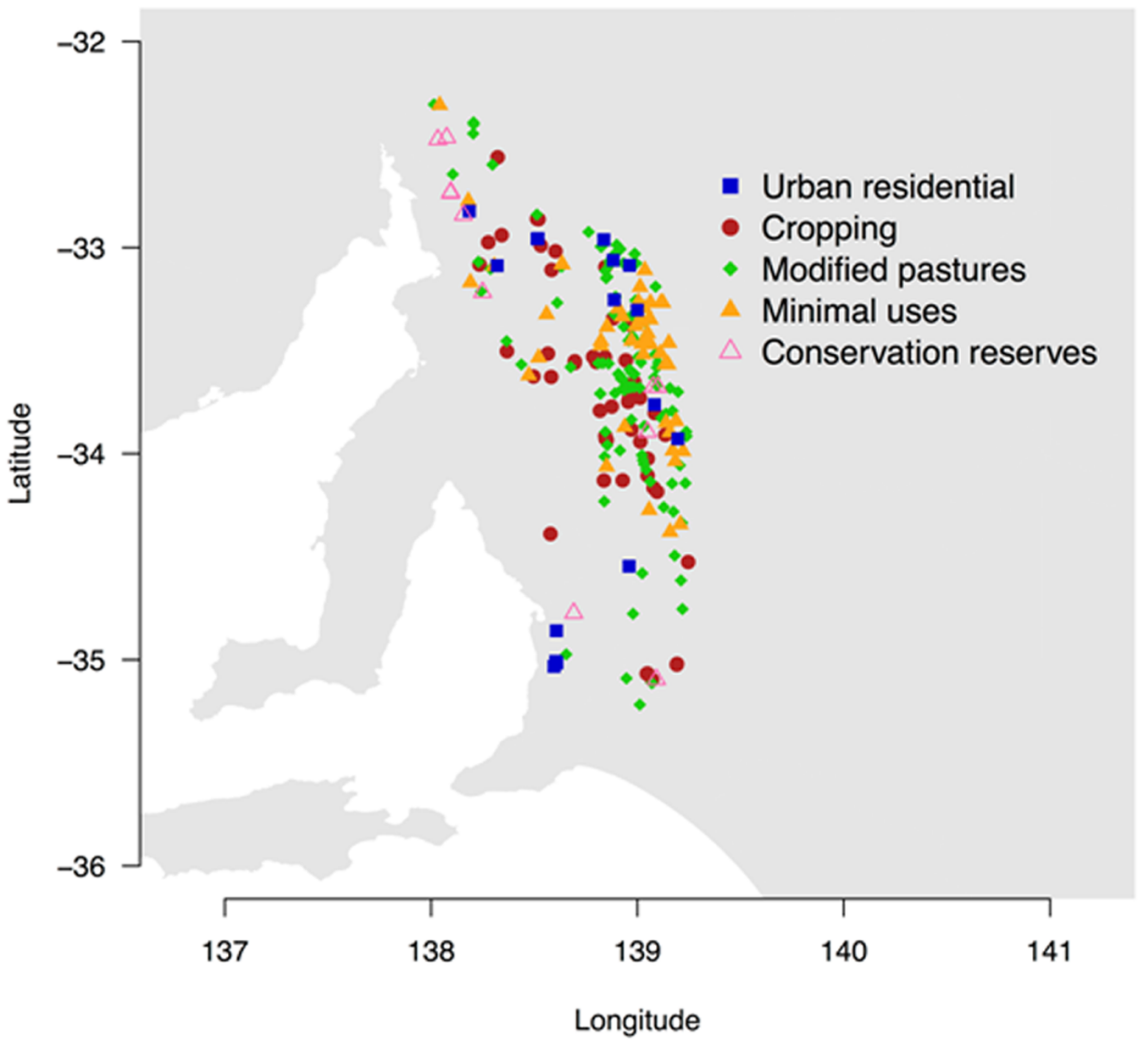
Map of the study area. **Dots represent grasslands surveyed in the Mount Lofty Ranges, South Australia**. Plots in grasslands associated within the following five different land-use classes were analysed following a gradient of disturbance: urban residential, cropping, grazing modified pastures, other minimal uses and native grasslands in conservation reserves.

The species diversity dataset consisted of 241 individual plots (typically 30 x 30 m) that were visited along the southern and northern Mount Lofty Ranges between February of 1991 and December of 1996. Plots comprised a gradient of land-use from heavily disturbed to relatively undisturbed areas (Fig 1).

Cover-abundance was recorded in the field employing a scale adapted from Braun-Blanquet [44], which included seven categories: N: not many (1–10) individuals; T: cover very small, sparsely or very sparsely present (less than 5%); 1: plentiful, but of small cover (< 5%); 2: any number of individuals covering 5–25% of the area; 3: any number of individuals covering 25–50% of the area; 4: any number of individuals covering 50–75% of the area; and 5: individuals covering more than 75% of the area. In order to provide an approximately metric variable for analysis [45,46], this scale was converted to percent cover as follows: N = 1%; T = 2%; 1 = 5%; 2 = 15%; 3 = 37.5%; 4 = 62.5%, and 5 = 87.5%.

For each plot, we computed Shannon diversity, species richness and cumulative cover, separating native from exotic species. Due to the known effect of seasonal rainfall on observed species richness and cover from one year to another in Mediterranean-type grasslands [47,48], we acquired data for total rainfall at each site during the twelve months prior to the sampling date using monthly precipitation layers (from March 1990 to December 1996) from The Ecosystem Modelling and Scaling Infrastructure Facility of TERN (ANUclimate layers [49]). Other long-term climatic variables, including mean annual temperature, fraction of photosynthetic active radiation, evaporation and water stress, as well as information about the nutrient status, including percent soil organic carbon, nitrogen and phosphorus, were obtained for each plot location from the Atlas of Living Australia (ALA; http://www.ala.org.au/).

We also incorporated information about the surrounding land-use of each plot from a 0.01° (approximately 1 km) resolution 'Catchment scale land use (ALUM secondary class)' layer [50], resulting in six different land-use classes corresponding with a disturbance gradient: (1) urban residential (heavy disturbance); (2) cropping (high disturbance); (3) grazing modified pastures (high-moderate disturbance); (4) grazing native vegetation (moderate-low disturbance); (5) other minimal use (includes natural areas on Defence land, stock routes, residual native cover and rehabilitation; low disturbance) and (6) nature conservation (nature reserves, National Parks and other protected areas; very low disturbance). Because there were only six plots within class (4), this was combined with class (6), which was considered to be the most ecological similar as very low disturbed grasslands.

### Data analyses

We conducted a Principal Component Analysis (PCA) including the climatic conditions of the sampled plots (*i*.*e*. precipitation in the year prior to the sampling date, mean annual temperature, fraction of photosynthetic active radiation, evaporation and water stress) and nutrient status (*i*.*e*. organic carbon, nitrogen and phosphorous), and extracted the first two components, PC1 and PC2, to represent the main, uncorrelated axes of environmental variation. The aim was to control for environmental influences rather than to determine the importance of particular variables.

To explore the effect of exotic species on Australian native species, we carried out partial correlation tests between species richness, cover and Shannon diversity for native versus exotic species groups. Partial correlations included PC1 and PC2 as covariates to control for the climatic conditions and the nutrient status of the plots and therefore explore relationships between both species groups without being affected by incidental correlations with environmental parameters, as species richness, for example, is expected to trend in both groups with rainfall [33,51]. We performed partial correlations considering all the surveyed plots together, and subsequently splitting by land-use to control for the degree of disturbance. All analyses were performed in R v3 [52], using the package ppcor v1.1 [53].

Differences in growth-forms represented by native and exotic species were visualised as percentages in categories (Muir Codes) scored in the BSSA method (Table 1).

**Table 1:** Muir codes for plant growth-forms recorded in survey plots (Department of Housing and Urban Development 1997).

Code	Growth-form
T	Trees > 30 m
M	Trees 15–30 m
LA	Trees 5–15 m
LB	Trees < 5 m
KT	Mallee (> 3 m)
KS	Low Mallee (< 3 m)
S	Shrubs > 2 m
SA	Shrubs 1.5–2.0 m
SB	Shrubs 1–1.5 m
SC	Shrubs 0.5–1.0 m
SD	Shrubs 0–0.5 m
P	Mat-plants
H	Hummock-grass
GT	Grass > 0.5 m
GL	Grass < 0.5 m
J	Herbaceous spp.
VT	Sedges > 0.5m
VL	Sedges < 0.5m
V	Vines (twiners)
MI	Mistletoes
X	Ferns
MO	Mosses, liverworts
LI	Lichens

## Results

**Fig 2:**
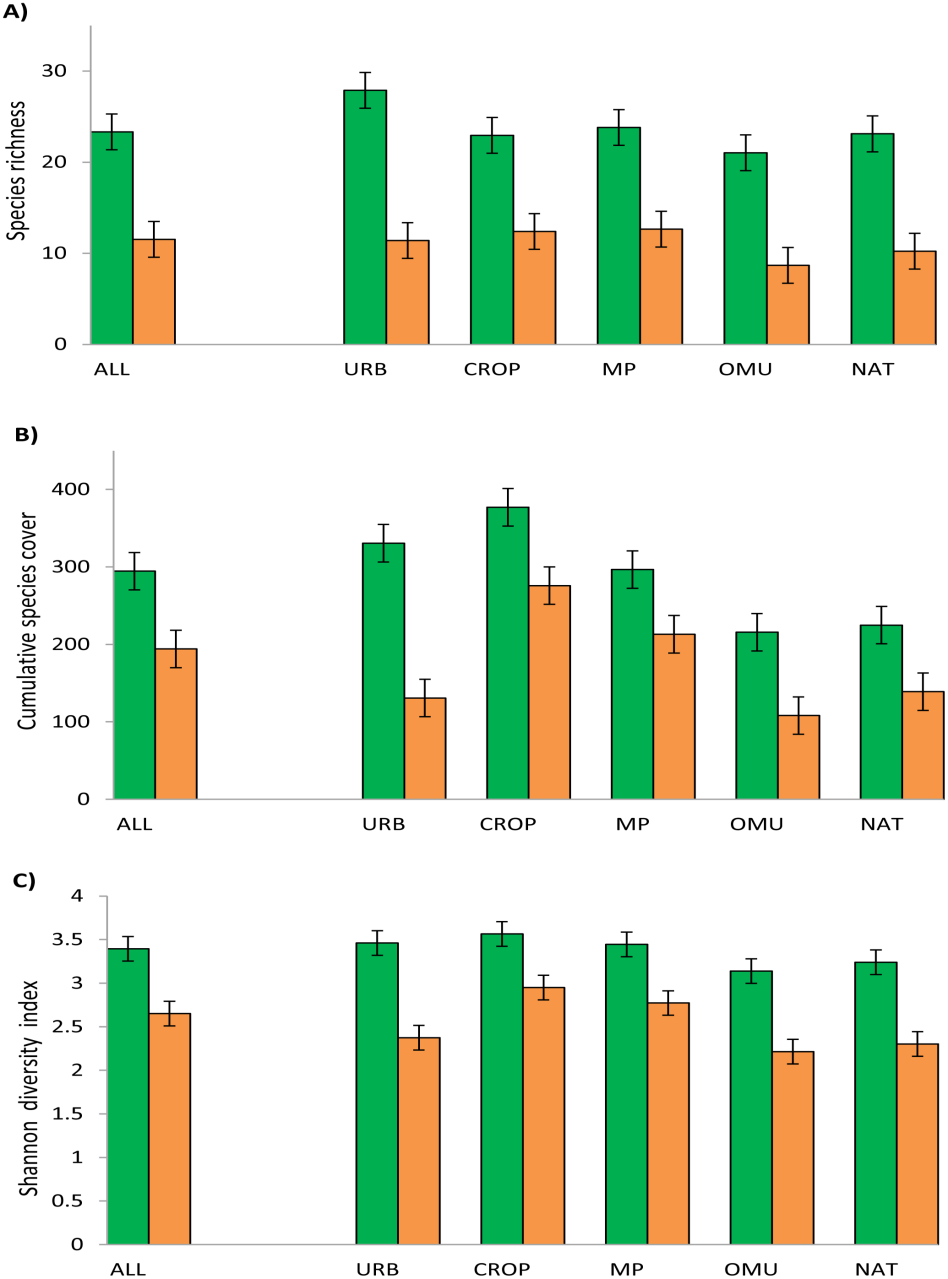
(A) Species richness; (B) cumulative species cover; (C) Shannon diversity; found in all the plots (ALL), and distinguishing by land use: Urban residential (URB), cropping (CROP), grazing modified pastures (MP), other minimal uses (OMU), and native grasslands in conservation reserves (NAT). Species were classified according to their origin, natives (green bars) and weeds (orange bars). The values correspond to the mean value (and standard error) considering the plot as the unit of analysis.

A total of 800 species were recorded in the dataset (Table 2; Fig 2A). Of these, 581 (72.6%) were Australian native, and 219 (27.4%) were exotic. Among the 241 survey plots, species richness and the number of plots within each land-use class varied considerably (Table 2; Fig 2A; see S1 Appendix in Supporting Information).

**Table 2:** Sample sizes and total numbers of species recorded within land-use classes.

Code	Land-use	Number of plots	Number of native species	Number of exotic species	Total number of species
All	All	241	581	219	800
URB	Urban residential	17	213	87	300
CROP	Cropping	51	280	128	408
MP	Grazing modified pastures	107	436	165	601
OMU	Other minimal uses	48	284	85	369
NAT	Native grasslands in conservation reserves	18	216	86	302

Native species cumulative cover and Shannon diversity were greater in heavily, highly and moderately disturbed grasslands (*i*.*e*. urban residential land-use, crops and grazing modified pastures, respectively) compared to minimally disturbed ones (*i*.*e*. other minimal land-uses and nature conservation reserves) (Fig 2B and 2C). Exotic species cumulative cover and Shannon diversity were greater in high-moderately disturbed grasslands, especially in crops and modified pastures, followed by urban, while grasslands associated with low-disturbance land-uses had the lowest presence of exotic species in terms of richness, cover and Shannon diversity index (Fig 2A–2C).

The first principal component (PC1) explained 45.2% and the second principal component (PC2) 19.2% of the environmental variation of the sampled plots. PC1 was positively correlated with evaporation (0.441), temperature (0.341) and fraction of photosynthetic active radiation (0.112), and was negatively correlated with organic carbon (-0.423), nitrogen (-0.347), phosphorous (-0.307), precipitation (-0.253) and water stress (-0.169). PC2 was positively correlated with precipitation (0.301), organic carbon (0.266) and water stress (0.193) and negatively correlated with phosphorous (-0.614), nitrogen (-0.558) and temperature (-0.327).

**Fig 3:**
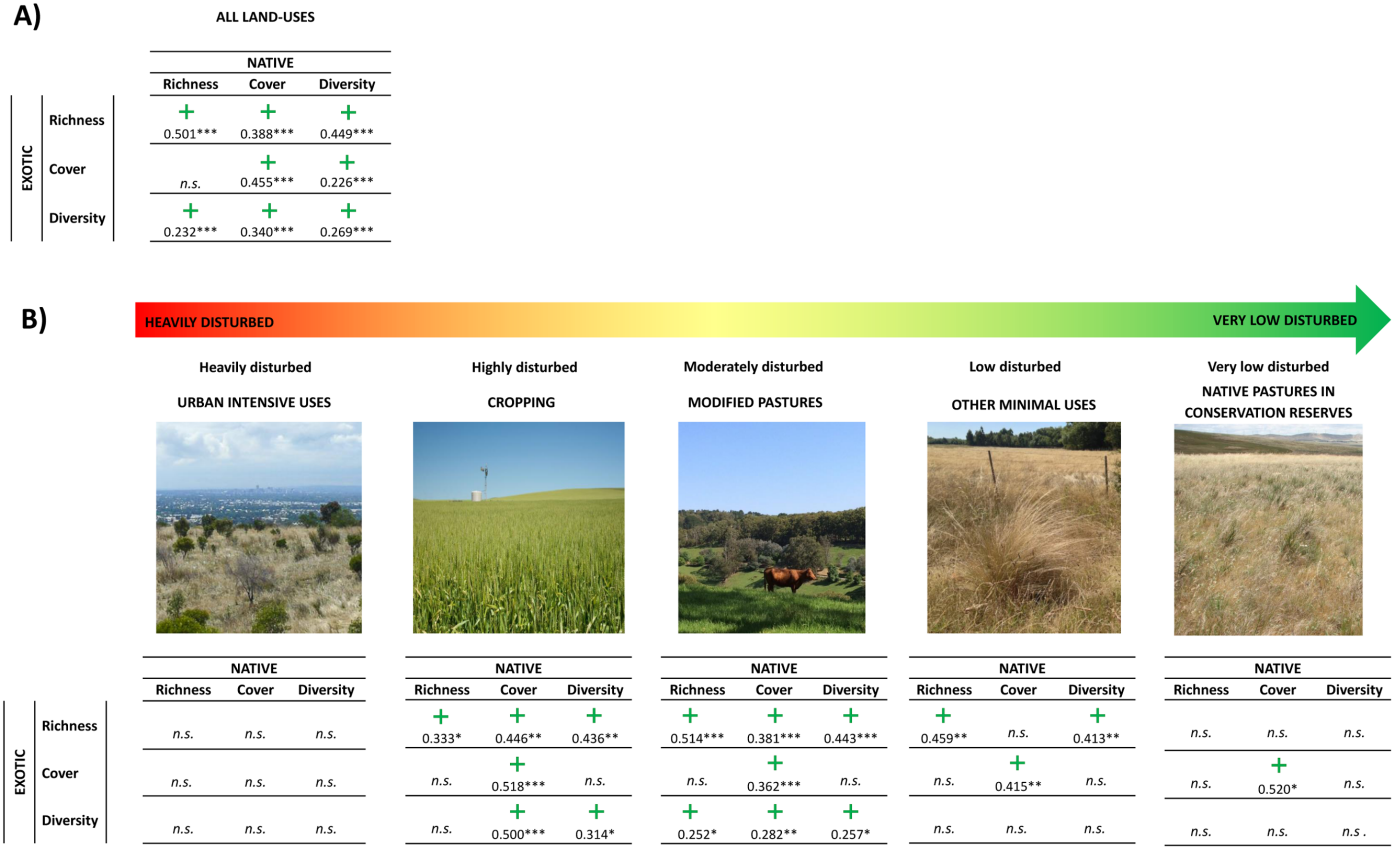
Correlations between native and weed species for values of species richness, cumulative cover and Shannon diversity. Correlations were calculated for (A) all the plots together and; (B) separating by land use. Only statistically significant interactions (p < 0.05) are detailed. Graphs are sorted following the gradient of anthropogenic disturbance: from heavily disturbed to undisturbed. Photos are illustrative only and do not represent sampled sites. Photo attributions (CC BY) from left to right: 1,2: Government of South Australia; 3,5: Greg Guerin; 4: Lawrie Conole c/o Atlas of Living Australia.

No negative relationships were found between natives and exotics in any case (Fig 3). When considering the 241 surveyed plots together, a positive significant relationship was found between both exotic species richness and exotic Shannon diversity with native biodiversity (in terms of richness, cover and Shannon diversity). Exotic species cover was positively related to native species cover and Shannon diversity, although no significant relationship was found with native richness.

When splitting the dataset, exotic—native relationships varied by land-use (Fig 3b). In grasslands located in urban residential areas, no significant relationships were found between exotic and native species. In high-moderately disturbed areas with cropping and grazing of modified pastures, many positive correlations were found between exotic and native species: exotic species richness was positively correlated with all of the native biodiversity parameters (richness, cover and Shannon index) and the cover of native and exotic species were also positively correlated. In low-disturbed grasslands that had minimal uses, exotic species richness was positively correlated with native species richness and Shannon diversity, and correlation between cover of exotics and natives was also significantly positive. In less disturbed native grasslands such as in conservation reserves, the only significant correlation was found between exotics cover and native cover.

**Fig 4:**
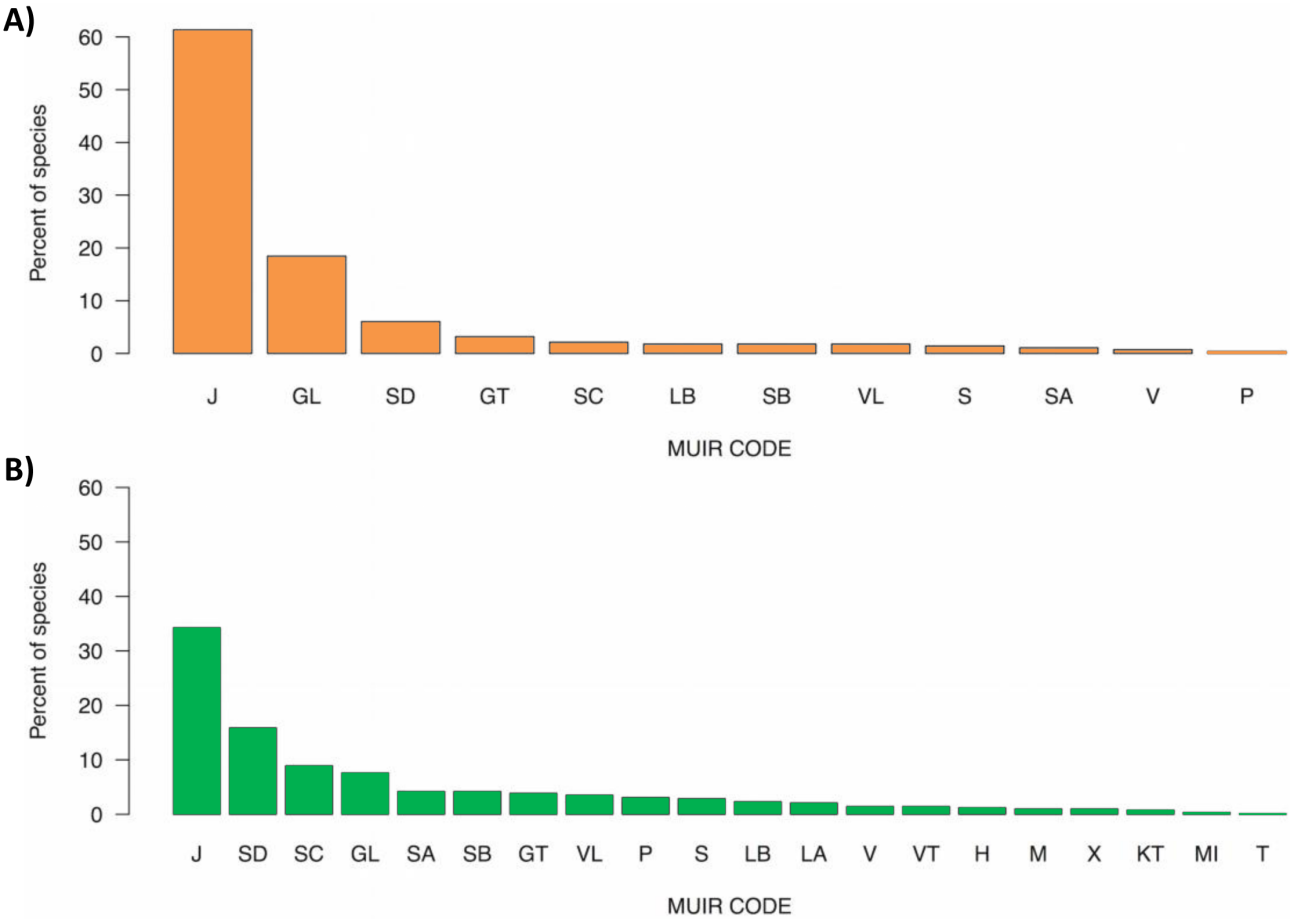
Percentages of recorded species in growth-form categories for (A) exotics; (B) natives. Growth-form codes are shown in Table 1.

Exotics and natives represented different spectra of plant growth-forms, whereby herbs and small grasses (mainly annual) were more frequent in exotics, and overall growth-form diversity was higher in natives (Fig 4).

## Discussion

The literature on exotic plant invasion contains mixed results on the question of whether native biodiversity is positively or negatively impacted [25,31,33], indicating that the invasion paradox is still unsolved, especially at larger-spatial scales. To some degree the outcome reflects the idiosyncrasies of particular invasive species, the suitability of the invaded habitat and the respective ecosystem response. Positive relationships between native and exotic species richness are generally expected at large-spatial scales because habitats with better environmental conditions and nutrient availability that are good for natives are also generally good for weeds–‘the rich get richer’ [54]. Nevertheless, ‘the rich get richer’ effect is negated by taking into account environmental variables and disturbance as controls, so the positive relationships reported in this paper necessarily indicate a different cause. Additionally, by testing not only for richness, but also for interactions from emergent, community-level properties such as cumulative cover and Shannon diversity, there is increased potential to understand whether more general rules emerge, and such empirical results form a basis for ecological models, experiments and theory to test specific mechanisms in more detail.

Our results show no apparent competition, or at least emergent negative effects, on diversity, between exotic and Australian native plant species in mediterranean-climate grasslands of South Australia. Instead, the positive correlations found between exotic and native biodiversity suggest that tolerance and/or facilitation processes are occurring between both groups of species. Contrary to the findings of recent studies stating that alien species established in disturbed landscapes impede the re-establishment of native species [2,55–57], the positive association existing between the cover of exotic and native species indicate a complementary role between exotic and native species in the occupation of space within this type of ecosystem.

While the dataset presented here concerns only general diversity metrics and cannot reveal causative processes, one explanation for positive exotic—native associations that would need to be tested further is the role of niche differentiation in which each species group has specialised strategies. For example, the exotic species in these grasslands contain a large proportion of ruderal species such as annual grasses (e.g. *Avena* spp., *Vulpia* spp., *Bromus* spp.) and herbs (e.g. *Hypochaeris* spp., *Trifolium* spp.), whereas the native species are mostly made up of perennial grasses (e.g. *Poa*, *Themeda*, *Austrostipa*, *Rytidosperma*), perennial herbs (e.g. *Leptorhynchos*, *Vittadinia*, *Wahlenbergia*) and woody species but also a diverse range of sedges, geophytes (e.g. *Arthropodium*) and ferns (Fig 4).

We detected positive interactions between exotic and native biodiversity more frequently in moderately disturbed grasslands associated with agrarian and farming activities. According to the eco-evolutionary model [34], weeds, mainly coming from the Mediterranean Basin, have been adapted to anthropogenic practices such as agriculture for millennia and are more prevalent in their introduced range under equivalent conditions [37]. The positive exotic—native associations under conditions of disturbance might be achieved through contrasting strategies: exotic species are mainly grazing-tolerant whereas native species are grazing-defensive [58,59]. In this sense, our results agree with previous studies which reported that an increase in vegetation cover associated with early colonisation by exotic species after a disturbance seems to create the appropriate conditions for the establishment of native species in Mediterranean grasslands of central Chile [31]. It seems therefore that in mediterranean-climate grasslands, exotic plant species co-evolve with anthropogenic practices in general and agrarian land-use in particular.

Our findings support a transition to a new paradigm, in which human perception about weeds should not be negative by default. We highlight the importance of adopting a more balanced view regarding exotic species diversity in general, understanding their presence in an invaded area as a holistic process [13]. Although problem weeds are sometimes targeted, conservation policies often promote the eradication of exotic plants regardless of identity [60]. Indeed, one metric used to assess the conservation value and condition of remnant ecosystems is the presence of weeds. A more nuanced approach to conservation may involve assessing exotic species in native systems on a case-by-case basis. Further studies evaluating specific interactions would elucidate mutualistic or negative interactions due to the presence of certain exotic species in an invaded range, although specific research is unlikely to occur for more than a handful of species.

From a conservation practitioner’s perspective, the findings reported here might be counter-intuitive as it is only natural to assume that highly abundant and visible ruderal weeds existing in a native community are having negative impacts. We found that in sites associated with moderately disturbing land-uses (cropping and grazing, but not urbanisation), native diversity was higher in the presence of exotics. This potentially challenges the notion that weeds need to be removed from remnant mediterranean-climate grasslands.

While the positive exotic—native diversity relationships came out strongly in this dataset, it is important to note that there are limitations to these conclusions. For example, we cannot discount the possibility that exotic—native diversity correlations may be different in grassland communities that are more or less completely modified from pre-European assemblages, or in other vegetation types that abound in the same region such as mallee, eucalypt woodlands and forest and shrublands, to name a few. It is also possible that the presence and cover of particularly competitive weed species may have negative consequences on native diversity that are not revealed when considering overall diversity across hundreds of survey locations. We also make no conclusion as to the potential for changes in native species composition in relation to weed diversity, rather than overall diversity. An extension to this work will be to test whether particular native species or functional groups fare better than others in the presence of high weed diversity. In other words, it remains to be tested whether positive or neutral diversity interactions between exotics and natives are upheld in terms of composition and function.

## Supporting information

S1 Appendix Dataset. Additional supporting information for this article may be found online.
(CSV)
